# Robust Lane-Detection Method for Low-Speed Environments

**DOI:** 10.3390/s18124274

**Published:** 2018-12-04

**Authors:** Qingquan Li, Jian Zhou, Bijun Li, Yuan Guo, Jinsheng Xiao

**Affiliations:** 1 State Key Laboratory of Information Engineering in Surveying, Mapping, and Remote Sensing, Wuhan University, Wuhan 430079, China; liqq@szu.edu.cn (Q.L.); JianZhou@whu.edu.cn (J.Z.); GuoYuan@whu.edu.cn (Y.G.); 2 College of Civil Engineering, Shenzhen University, Shenzhen 518060, China; 3 School of Electronic Information, Wuhan University, Wuhan 430072, China; xiaojs@whu.edu.cn

**Keywords:** lane detection, symmetrical local threshold (SLT), Bresenham line voting space (BLVS), Kalman filter

## Abstract

Vision-based lane-detection methods provide low-cost density information about roads for autonomous vehicles. In this paper, we propose a robust and efficient method to expand the application of these methods to cover low-speed environments. First, the reliable region near the vehicle is initialized and a series of rectangular detection regions are dynamically constructed along the road. Then, an improved symmetrical local threshold edge extraction is introduced to extract the edge points of the lane markings based on accurate marking width limitations. In order to meet real-time requirements, a novel Bresenham line voting space is proposed to improve the process of line segment detection. Combined with straight lines, polylines, and curves, the proposed geometric fitting method has the ability to adapt to various road shapes. Finally, different status vectors and Kalman filter transfer matrices are used to track the key points of the linear and nonlinear parts of the lane. The proposed method was tested on a public database and our autonomous platform. The experimental results show that the method is robust and efficient and can meet the real-time requirements of autonomous vehicles.

## 1. Introduction

As an effective method to improve the transportation efficiency and reduce the risk of road accidents, automatic driving has been developing rapidly. Vision-based lane detection algorithms not only provide lane departure warning (LDW) to advanced driver-assistance systems (ADAS), but also guide autonomous vehicles to drive along the ego-lane by themselves. Lane detection algorithms developed from early experimental prototypes like AutoVue [1] and GOLD [2,3] to various commercial applications. A standard configuration of an lane departure warning systems (LDWS) can be broadly divided into three parts: Lane detection, lane tracking and lane departure [4]. Sequences of road images are captured by an onboard-camera high up on the windshield, and lane departure alerts occur to protect the driver from some unintended dangerous driving behaviors. Most of these products are designed for highway and freeway driving, where the illumination conditions and geometric models of the road are simple. However, to expand the coverage of intelligent transport systems, lane detection systems should also work robustly on local low-speed roads. 

Compared with the high-speed structural road environment, road shapes in local low-speed environments are complex and variable. Complex environments are challenges for the real-time and robustness requirements of autonomous vehicles and robots. Many proposed methods have attempted to solve this problem by using light detection and ranging (LIDAR) [5,6,7,8], stereo cameras [9,10,11], or multiple sensors [12]. Typical characteristics of the low-speed road are as follows: The speed limit of the road is under 30 km/h; the width of the road is narrow; the geometrical model of the road is diverse. Besides, the number of lanes in low-speed roads are less than four which means that usually a continuous solid line exists on one side of the ego-lane. According to the differences of the road surface, the low-speed environment can be divided into two types in some developing countries like China: Structure road with well-covered lane markings on the cement or asphalt surface, unstructured road without lane markings with pit or rut on sand or dirt road. The proposed method provides a robust lane detection method for the first low-speed environment based on a monocular camera. There are two main advantages of using a monocular camera: It is inexpensive and easily meets real-time requirements, and provides dense information about the environment.

Although many lane detection methods have been proposed for different road environments, the robust and real-time are the two most important requirement of the algorithms [13]. As the conclusion proposed by Navarro [14], partial lane departure or reliable warnings are more effective and acceptable by drivers than full lane departure or unreliable warnings. In this paper, in order to ensure the robustness of the proposed method, edge points of the lane markings will be extracted from high resolution images which is up to 1920 and 1200 pixels in a horizontal and vertical direction. Then, an improved symmetrical local threshold (SLT) [15] edge extractor with accurate marking width limitation is introduced to position the edge points in independent rows of the image. Although the high-resolution images add the computation of the algorithm, the Bresenham line voting space (BLVS) proposed in this paper is insensitive to the increased pixels of the image and can provide a real-time line segment detection method. In addition, there are multiple possibilities for the geometric construction of lane markings, such as straight lines, polylines and curves. The geometrical model of the lane markings will be described as piecewise functions. Corresponding to the geometrical model function, marking points in linear and nonlinear parts will be tracked with different Kalman flters.

The main contributions of this paper are as follows. (1) An improved symmetrical local threshold feature extraction method with high-accuracy width limitation is proposed to make sure that the position of the lane markings can be extracted exactly and robustly. (2) In order to accurately extract line features from discrete feature points in real time, a novel Bresenham line voting space (BLVS) is proposed. (3) Geometric model selection and piecewise geometric fitting are proposed to ensure that the shape of the road is precisely described. (4) Finally, to adequately utilize the spatial correlation information, we adopted a Kalman filter specific to the linear and nonlinear parts with different status vector and transfer matrix.

This paper is organized as follows. Section 2 presents related work on lane detection methods. Section 3 presents an overview of our method. The proposed feature extraction detection method, including edge point extraction, line segment detection, and validity of results, is explained in Section 4. In Section 5, we introduce a multiple model-based geometric fitting method for all line segments. Tracking the parameters of the global geometric model are provided in Section 6. Finally, the experimental results and a detailed performance evaluation are described in Section 7.

## 2. Related Work

Various lane detection methods have been developed for simple and complex environments. Most lane detection methods can be decomposed into the following three steps: Feature extraction of lane markings is first performed, including edge point and line segment extraction. Color, gradient, and intensity are three typical characteristics of lane markings. Sobel [16], Canny [17], Steer filter [14] and Garbor [18] filters are gradient-based and intensity-based edge point extraction methods. Based on the edge information extracted by these detectors, Hough transform [19,20,21,22], line segment detector (LSD) [23], edge drawing line detector (EDLines) [24] and fast line detector (FLD) [25] are then used to find straight lines in the image edge. The maximally stable extremal regions (MSER) method was proposed by Küçükmanisa et al. [26,27] to solve lane detection problems in a complex environment, such as shadows or at night. Finally, some deep learning-based methods [28] have been proposed to extract lane features in end-to-end environments, which have achieved higher accuracy than traditional methods.

Estimating the geometric models of lane markers is another important component of lane detection. Different kinds of post-treatments have been proposed to perform these estimations. In previous work, this step was performed by using a straight line model to simplify highways and freeways [29]. In addition, interpolating curves such as splines [30,31,32] are popular geometric models for estimating lane markings in local low-speed road environments. However, control points along the spline are often difficult to determine. In addition, polynomial curves [33] are used in some low-curvature environments. However, second-order curvature may be influenced by some error features. Therefore, some studies have proposed an error punishment adjustment to enhance the robustness of the fitting process [34]. 

Finally, tracking the parameters in the geometric model is a key component for enhancing the robustness of the algorithm. Kalman filtering is widely used in this step [35,36,37]. Some authors [29] proposed a parallel assumption about both sides of the road lane. The width of the lane, angle of deviation, and lateral offset between the middle of the lane and the projected center of the camera were used to construct the status vector of the car. However, the lack of consideration of curvature makes this method unsuitable in a curved environment. Nieto et al. [38] efficiently designed and implemented a particle filter by separating the state into linear and nonlinear parts.

## 3. Overview of Our Method

**Figure 1:**
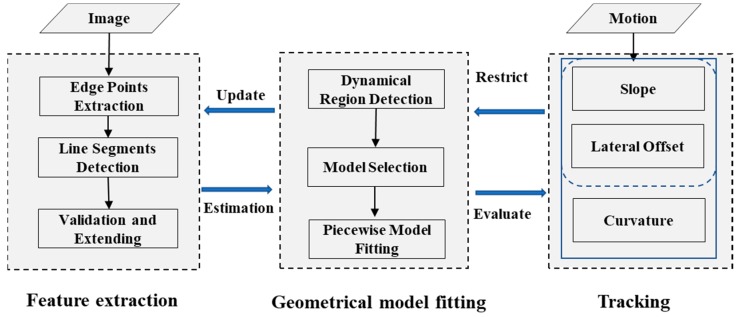
Overview of our lane detection method.

This paper proposes a robust and effective real-time lane detection method consisting of three parts: Extracting lane markings, estimating a geometric model, and tracking the key points of the geometric model. In our method, high-accuracy lane markings pixel widths were calculated before being extracted in independent image rows. The robustness of the edge detector is ensured by gathering the pixel width constraints along with the symmetrical characteristics of the gradient between both sides of the lane marker based on the symmetrical local threshold (SLT) method. Serial local-adaptive-detection windows from near to far were designed to detect line segments step-by-step. A novel BLVS was introduced to enhance the computational efficiency of the method. Then, the validity of the line segment was checked by the line validation method proposed by Akinlar et al. [21]. After all local regions were processed, we used a model selection function to choose an appropriate geometric model to piecewise fit the shape of the road. Finally, different kinds of status vectors were used to track the linear and nonlinear parts of the road model using Kalman filter. An overview of our method is shown in Figure 1.

## 4. Feature Extraction

The proposed method starts by extracting robust and discriminative features from road images. As lane markings comply with specific standards in most developed countries, with a fixed width of bright lines or curves on dark backgrounds, the dark–bright–dark pattern of lane markings is widely used. Some studies used a two-dimensional Gaussian kernel containing a smoothing Gaussian and a second-derivative Gaussian to filter an inverse perspective mapping image [39], but errors commonly occurred in distinguishing road markings and curbs. Another proposed method [12] used the width of the lane markings to construct a symmetrical local threshold (SLT) edge detector, and concluded that extractors like symmetrical local threshold (SLT) produce the best result in most cases. However, the method used for estimating the width of lane markings in that paper is unstable and sensitive to variability in the pitch angle of the camera. When the environment is complex and contains shadows, bright spots, and other challenging illuminations, the poor accuracy of the widths of lane markings cannot prevent the extractor from disturbances due to complex illumination.

An improved method based on symmetrical local threshold (SLT) is proposed in this paper to provide effective and robust edge detection by adding an accuracy width constraint. In order to accurately estimate the local linear relationship between the widths of lane markers in road and image planes, the region of interest (ROI) is first initialized in the bottom of the image by considering the vibration of the vehicle.

### 4.1. ROI Initialization

**Figure 2:**
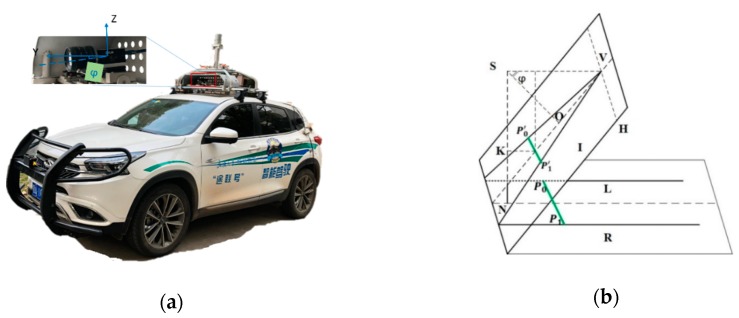
(**a**) Position of the camera on the vehicle. (**b**) Pin projection model of monocular camera between image plane I and road plane R. The focal length of the camera is described as the distance between project center S and optical center O. Pairs of edge points on both sides of the lane, marked L and R, can be described as $P_{0}$ and $P_{1}$ on the road plane and $P_{0}^{\prime }$ and $P_{1}^{\prime }$ on the image plane, respectively. The distance SN is the height of the camera. The pitch angle of the camera is φ and V is the vanishing point of L and R. SK is the projected length of the distance from the middle of $P_{0}^{\prime }$ and $P_{1}^{\prime }$ to V.

To obtain accurate pixel widths of lane markers in the near-vehicle region, a popular assumption that the road is approximately planar is used in this Section. The transformation of the widths of lane markings between the image plane and road plane can be set up by the geometric model of the camera, as shown in Figure 2b. We started by defining a world frame centered at the camera’s optical center, a camera frame, and an image frame, as shown in Figure 2a. The right side of the lane marking is the shifted version of the left side of the road with distance *W* along the geometric model of the lane marker. After projecting from the ground plane to the image plane, the distance *S* between both sides of the lane marker can be calculated by a linear model, as shown in Equation (1):

(1) $$s=\frac{W}{H}cos\varphi \left(v_{y}-y\right),$$

where *W* is the shift distance between $P_{0}$ and $P_{1}$. *H* is the height of the camera (SN). $v_{y}$ is the vertical coordinate of the vanishing point *V*, and *y* is the vertical coordinate of the lane marking.

However, the pitch angle of the camera dynamically changes when the autonomous vehicle is moving on the road. The variety of camera postures unavoidably break the linear relationship between *S* and *W*, which leads to the failure of the constraint of the width of the lane markings, especially in far regions. Therefore, we chose an error model to analyze the deviation of *S*, as follows.

The relationship between the vertical coordinates of the vanishing point and pitch angle of the camera can be described with Equation (2):

(2) $$\tan\varphi =\frac{v_{y}}{f},$$

where *f* is the focal length of the camera. Then, Equation (1) can be changed to a function of pitch φ:

(3) $$S=\frac{W}{H}cos\varphi \left(f\times tan\varphi -y\right).$$

The error model of the pitch angle based on Equation (3) can be described as:

(4) $$\delta \left(S\right)=\frac{W}{H}\left(fcos\varphi +ysin\varphi \right)\times \Delta \varphi ,$$

where Δφ is the range of the pitch angle. In order to distinguish a wider noise marking that is ζ times the width of the true lane marking, the row number of the marker point in the image should meet the condition:

(5) $$S_{max}\;<\;S_{min}^{\zeta },$$

where $S_{max}$ is the maximum width of the lane marking and $S_{min}^{\zeta }$ is the minimum width of the wider noise marking. An ROI selection method based on the error model is proposed to produce a robust region near the vehicle under the minimal row number $R_{min}$ in the image to meet the condition in Equation (5), as Equation (6) shows:

(6) $$R_{min}=\frac{fsin\varphi -lfcos\varphi \Delta \varphi }{lsin\varphi \Delta \varphi +fcos\varphi },$$

where

(7) $$l=\frac{\zeta +1}{\zeta -1}.$$

### 4.2. Edge Detection

**Figure 3:**
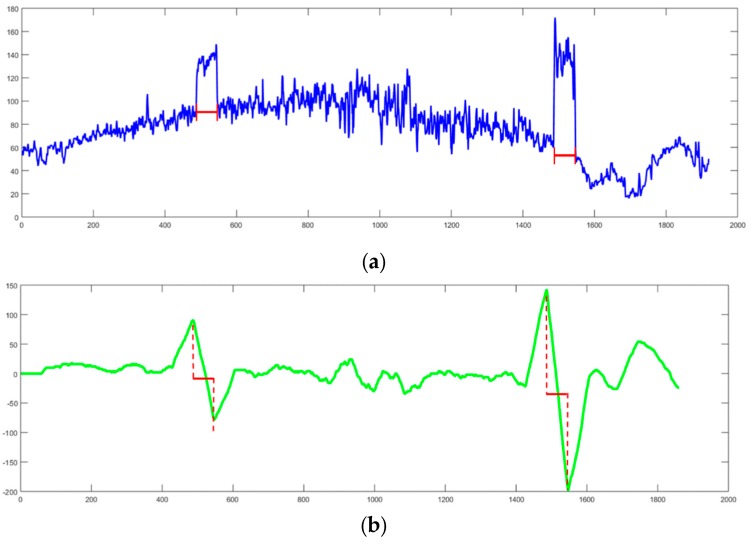
(**a**) Raw intensity of a row of the image. (**b**) Result of gradients corresponding to the input row of the image and the red line present that the pixel width of the peak and valley is equal to the widths of lane markings.

To accurately locate the edge points of the lane markings in the image, the limited width and the symmetrical positive-negative gradient were introduced. Our idea was to use adaptive slider window sizes in which the width was the same as the lane marking. The gradient of each pixel was calculated with the help of the slider window. Then, pixels with pairs of peak and valley gradients were selected as candidate edge points of lane markings. Consider a vector I that describes a row in the images between [*j_min_*,*j_max_*] and the gradient E within limited width, as described by Equation (8):

(8) $$E_{j}=\{\begin{array}{c} \frac{1}{S}\overset{L}{\underset{k=1}{\sum }}I_{j+k}-I_{j-k}\;j=j_{min}\\ E_{j-1}+\frac{1}{L}\left(I_{e}-I_{m}\right)\;j\in \left(j_{min},j_{max}\right] \end{array}$$

where *E_j_* designates the gradient and *S* corresponds to the lane width estimation centered around *j*, the pixel abscissa on a row. The result of the gradients of an independent row is shown in Figure 3. 

**Figure 4:**
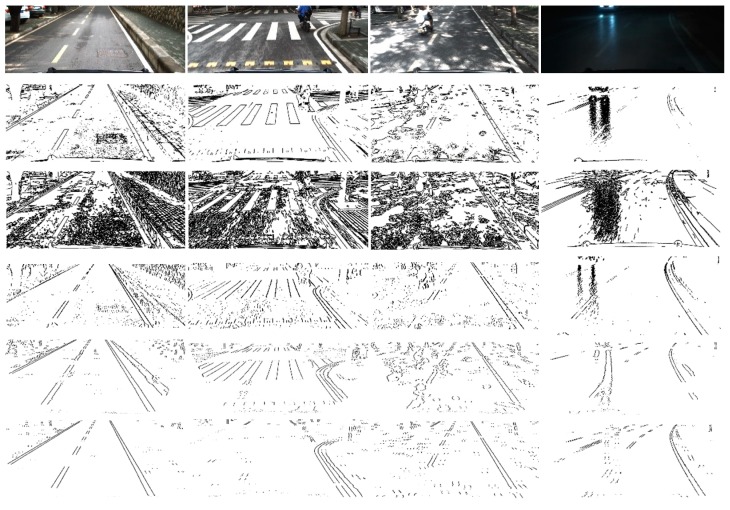
The first row shows the scenarios with straight line, intersection, bright spot and night. The results of edge extraction from the second row to the last row are extracted by Sobel, Canny, Steer Filter, symmetrical local threshold (SLT) and the proposed method.

The Figure 3a is the histogram of raw intensity of a row in the image, and the Figure 3b is the result of positive-negative gradient that calculated by Equation (8). Compared to the raw intensity, the result of the edge detection method is smooth and contains obvious peak and valley features with the same pixels width with lane markings which is helpful to distinguish lane markings from complex road background. The serious slider window that was designed by Equation (8) will be used to calculate the mean gradient of the pixels. When the slider widow is on the left edge of the lane markings, the positive gradient gets a local maximum value and a local minimum value will be calculated on the right edge of the lane markings. If the absolute values of gradients of a pair of peak and valley points are bigger than a given threshold and the pixel width between them is near the width of lane markings, both of them will be realized as the left and right edge of a lane marking. Figure 4 shows the edge extraction results of some popular methods like Sobel, Canny, Steer Filter, symmetrical local threshold (SLT) and the proposed method in four different road scenes containing: Straight road, intersection, light spot and night.

Compared to the changeable lighting conditions like bright spot, shadow, night and so on, the width of the lane markings is relatively constant. As it shows in Figure 4, the proposed method can not only extract the lane markings exactly, but also prevent the extraction result from the disturbance made by the crack in the middle of the lane markings, zebra in the intersection, light spot under the trees and reflect light on the road in night. 

This method has three major advantages: (1) The symmetrical positive-negative gradient extractor is robust and effective; (2) the extractor is insensitive to the noise caused by shadows, bright spots, and traffic signs on the ground; and (3) the position and width of lane markings corresponding to rows of the image can be accurately calculated, which is helpful for adjusting the linear relationship between the width of the lane marker and vertical coordinates in the image plane.

### 4.3. Line Segment Detection

This stage involved detecting lane segments from the edge points in a local region. There are two main steps in this part: Using a novel BLVS to detect lane segments in road plane, followed by a least squares-based validity function to remove noise points. With the help of the perspective projection model between the camera and the road plane, the positions of edge points of lane markers are projected to a grid on the road space. The middle line of the lane is described as two points that are located on the top and bottom edges of the grid. Line segments in the grid can be uniquely described as the offset from the left-bottom point and the abscissa deviating between these end points.

#### 4.3.1. Bresenham Line Voting Space 

Several methods have been proposed for line segment detection in binary images, such as Hough Transform [19,20,21,22], line segment detector (LSD) [23], edge drawing line detector (EDLines) [24] and fast line detector (FLD) [36]. As the candidate edge points extracted in the last section are nonadjacent in the road plane, Hough Transform was first tried to select the line segments. The discrete coordinates of the edge points in the image plane were transformed to float slope parameters and integer distances in Hough space. Although this step generally benefits extracting line segments from discontinuous edges, the huge number of floating computations reduced the efficiency of the algorithm and resulted in precision loss in far regions.

In order to increase the efficiency of the algorithm to meet the real-time requirements, Bresenham Line Voting Space (BLVS) is proposed in this paper. This method can be decomposed into three steps: Initializing the lookup table of the region around the vehicle, selecting the line segments in the local BLVS, and selecting the candidate line segments.

**Figure 5:**
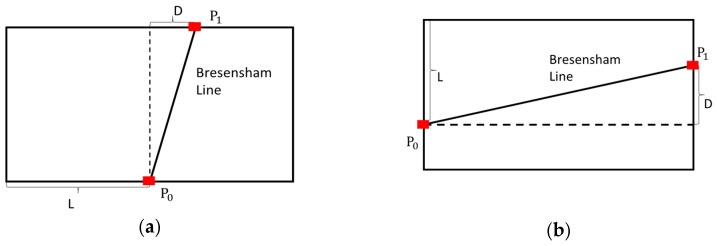
Vertical (**a**) and horizontal (**b**) line segments described in Bresenham line voting space (BLVS). L is the lateral displacement of end point *P*_0_ at the bottom edge of the space and D is the deviation from *P*_0_ to end point in the top edge *P*_1_.

**Figure 6:**
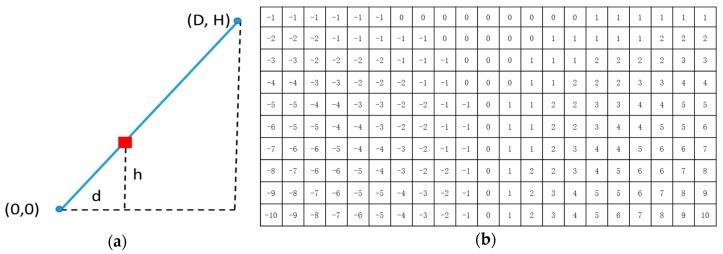
(**a**) The process of calculating lateral displacement d with step h of the lookup table by generating a Bresenham line from (0,0) to (D,H). (**b**) An example of lookup table in which D belongs to [–10, 10] and height is 10.

Different from Hough Transform, the parameters of the line segments in BLVS can be described by two discrete values. In order to uniquely define all the line segments within the detection grid, the line segment types were classified as vertical and horizontal according to the parameter of the slope. When the absolute value of the slope is bigger than one, the vertical BLVS is used. As shown in Figure 5a, all the possible line segments in the discrete grid can be defined by pairs of points from the top and bottom edges of the grid. Parameters of the line segments can be described as L and D, which mean lateral displacement of *P*_0_ and horizontal deviation of *P*_1_ from *P*_0_, respectively. The horizontal BLVS is used to choose horizontal line segments whose slope is smaller than one. The displacement parameters of the line segments are similar to vertical BLVS. After generating a Bresenham line between *P*_0_ and *P*_1_, we obtained all the possible points on the line segment. When the range of D and the height *H* of the grid are defined, all possible line segments across the point in the detection grid with vertical and horizontal coordinates can be calculated by a displacement lookup table, as shown in Figure 6.

When the ranges of D and H of the detection grid have been determined, the cells of the lookup table record displacement information is used to calculate pairs of L and D to find all possible line segments that would cross the marker point. As shown in Figure 6, the lookup table can be initialized by generating a Bresenham line segment from bottom to top. The displacement value is calculated and saved step-by-step. Figure 6b depicts an example of the lookup table, which has initialized the deviation parameter D, which belongs to [–10, 10], and the height H of the table is 10. 

The BLVS algorithm generates a corresponding voting table for the local detection region. The height of the voting table is the same as the number of scan lines, and the width is defined by the direction constraint of the local region. During the real-time process of the algorithm, the voting table for a local region can be quickly calculated by the Bresenham line algorithm.

**Figure 7:**
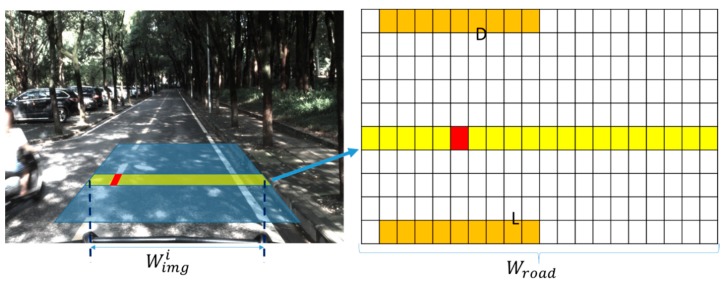
Edge point (red) belonging to a row of the detection region (yellow) chooses the candidate line segments (dark yellow).

**Figure 8:**
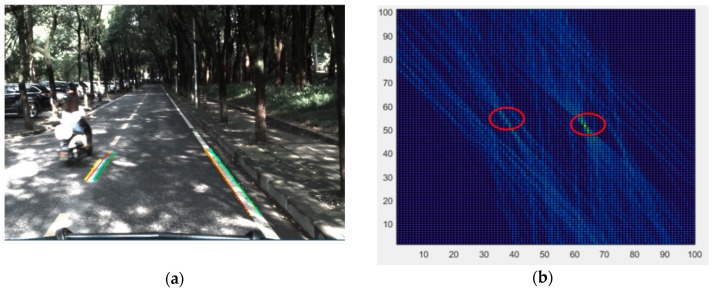
(**a**) Result of line segments and (**b**) corresponding selection table. The two peak points in the red circle represent the left and right lane markings of the ego-lane.

All the candidate edge points of the local trapezoid region on the image plane are projected to the rectangular grid on the road plane. The transformation process can be described by Equation (9). The process of choosing the line segments in a local region is depicted in Figure 7. As shown, all the possible line segments in the space are uniquely defined by the two end points on both sides of the up-and-down edge. If the edge point *P(i,j)* belongs to the local detection region, all the parameters of the line segments that cross the point are calculated by adding the horizontal coordinate *j* with the value of row *i* of the table. The result of choosing the line segments in the local region is shown in Figure 8.

(9) $$J_{road}=\frac{W_{img}^{i}}{W_{road}}\times \left(J_{i}-J_{i}^{0}\right),$$

where $J_{i}$ is the horizontal coordinate of the edge point in the ith row, $J_{i}^{0}$ is the start point on the left, $W_{img}^{i}$ is pixel width of the detection region in the ith row, and $W_{road}$ is the width of the detection grid.

#### 4.3.2. Line Segment Validation

**Figure 9:**
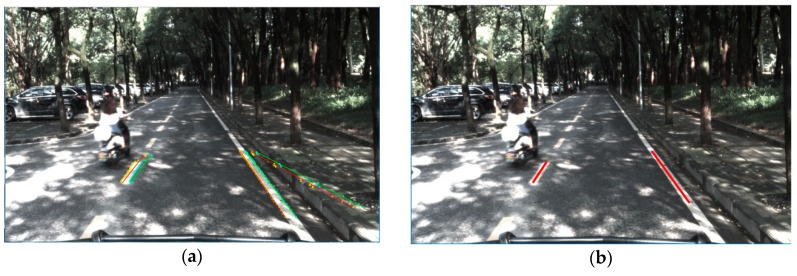
(**a**) Line segments voted by the BLVS and (**b**) the middle line of the lane marking after line segment validation.

Although the candidate lane segments were extracted in the grid, some noise points were also present. A line segment validation method proposed by Akinlar et al. [21] was used to remove noise feature points based on the assumption that lane markings on the ground are well covered. First, the minimum length of a single lane marker was constructed from the farthest feature points of the lane segment. Least squares was used to calculate the parameters of the fitting line and their residual error. When the residual error is smaller than a given threshold, the minimum length of feature points is realized as the most robust segment among all the feature points. Based on the parameters of the minimum line segment, other feature points belonging to the same segment are confirmed. The result of the line segment validation is shown in Figure 9.

## 5. Geometric Parameter Fitting

The boundary of the lane can be considered a sequence of piecewise curves or straight lines. As the shape of the road must comply with the soft requirements of driving, which the curvature along the road must change slowly and be larger than the minimal turning radius of the vehicle. We assumed that the lane segment near the vehicle can be realized as a straight line. Therefore, the geometric model can be described as straight line at the bottom of the image plane.

Based on the candidate line segments detected in the near region, a dynamic detection grid was constructed with the help of the geometric parameters of the near lane. Pixel widths of the lane markings in the next region can be calculated by the linear relationship between the pixel width and rows of the image in previous region. The next detection grid corresponding to the candidate lane considered the geometric model of the current road. When the road is straight, the extended grid can be simply defined by a linear extension of the candidate lane. If the shape of the road is polyline, curved, or another complex shape, the turning radius and the type of candidate line are considered. 

**Figure 10:**
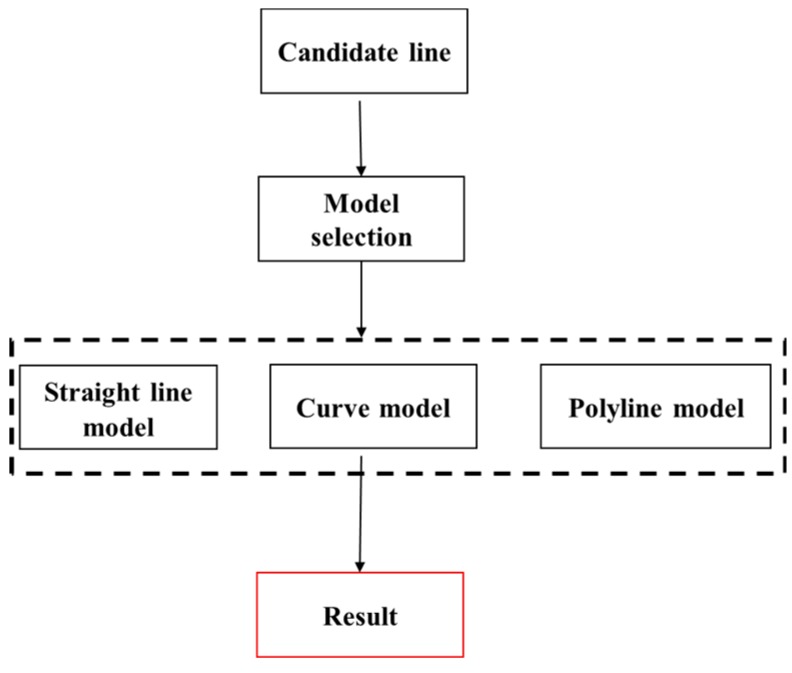
Flowchart of geometric model fitting.

Figure 10 shows a flowchart of the model selection of our method. The type of candidate lane was considered first because the solid lane was continuous. First, the straight model was applied to probe the far region where the shape of the road was straight. Then, the curve model was used when the slope deviation between the adjacent line segments expanded the given threshold in the first step. A second-order curve model initialized from the end points of the candidate line segments was used to fit the feature points in the far region. When the residual error of the curve model met the continuous condition, a larger range of detection beginning with the end of the curve was created to ensure that all possible lane markers were included. Efficiency in this condition is lower, but the speed of the vehicle must be reduced at the same time. 

In addition, when the ground truth of the road is polyline, the slope deviation between the adjacent line segments would be similar to the curve model, but the second-order curve-fitting model would fail. Therefore, the process of line segment validation was applied when the curve model failed. Then, another straight line model was initialized in the far region when the shape of the road was polyline.

## 6. Tracking of the Model

The link points between adjacent line segments are key points of the whole geometric model, which not only distinguish the shapes of line segments but also enhances the accuracy of model tracking. Although the geometric fitting of lane segments over the entire road scene provides a mathematical representation of the shape of the road, tracking the geometric parameters further improves the robustness of the detection results. However, two main kinds of noise disturbed the accuracy of the tracking process: The divergence between the geometric model and the real shape of the road, and the poor quality of the estimation of the vehicle motion. The solution proposed in this paper was to track the key points of the global geometric model, which are insensitive to errors of the local feature points and robust to variable changes in the road scene.

As key points in different parts of the geometric model have different motions, a tracking model of the key points would be composed of linear and nonlinear parts. In the linear part, status vectors of the key points would include horizontal and vertical coordinates ($x_{r,t},\;y_{r,t}$) with variations described as ($\Delta x_{r,t},\;\Delta y_{r,t}$). Then, a linear Kalman filter was used to track parameters of key points, and the transfer matrix can be described by Equation (11).

(10) $$x_{t}=\left(x_{r,t},\;y_{r,t},\;\Delta x_{r,t},\;\Delta y_{r,t}\right).$$

(11) $$A_{linear}=\left[\begin{array}{cccc} 1 & 0 & 0 & 1\\ 0 & 1 & 0 & 1\\ 0 & 0 & 1 & 0\\ 0 & 0 & 0 & 1 \end{array}\right].$$

**Figure 11:**
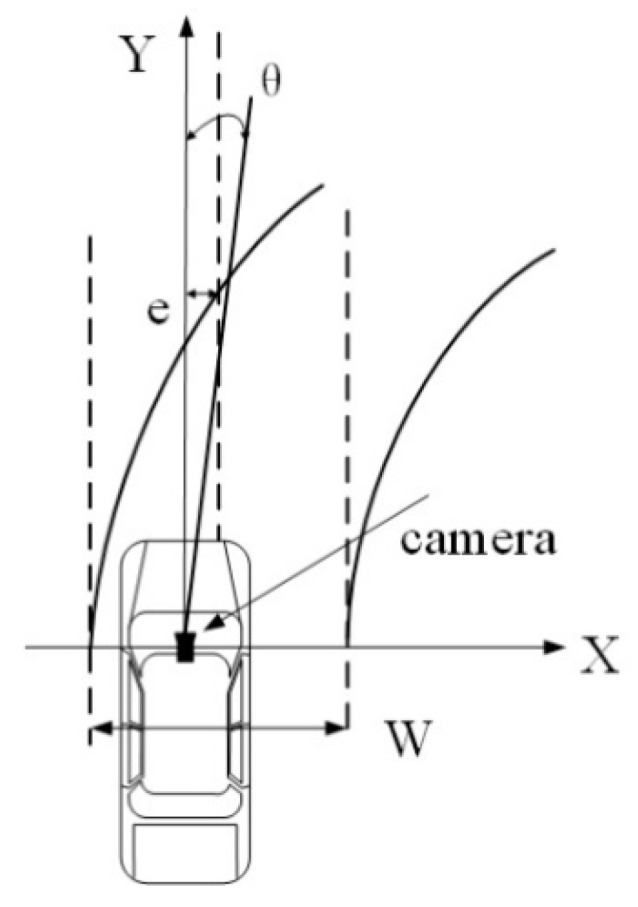
Curve model of the road.

In order to track the key points belonging to the nonlinear segments, a curve model of the road proposed by Watanabe et al. [29] was used. Figure 11 shows the curve model of the road, where the width of the ego-lane W, the lateral displacement between the center of the camera and the center of the ego-lane $e_{t}$, the yaw angle $\theta_{t}$ of the lane to the *y*-axis in the road plane, and the curvature of the lane were used in the status vector, as shown in Equation (12). In addition, the corresponding transfer matrix is described by Equation (13).

(12) $$x_{t}=\left(W_{t},\;e_{t},\;\theta_{t},\;c_{t}\right).$$

(13) $$A_{nonlinear}=\left[\begin{array}{ccccc} 1 & dz_{t} & \frac{1}{2}dz_{t}^{2} & 0 & 0\\ 0 & 1 & dz_{t} & 0 & 0\\ 0 & 0 & 0 & 1 & 0\\ 0 & 0 & 0 & 0 & 1 \end{array}\right].$$

## 7. Experiment Using Our Method

**Figure 12:**
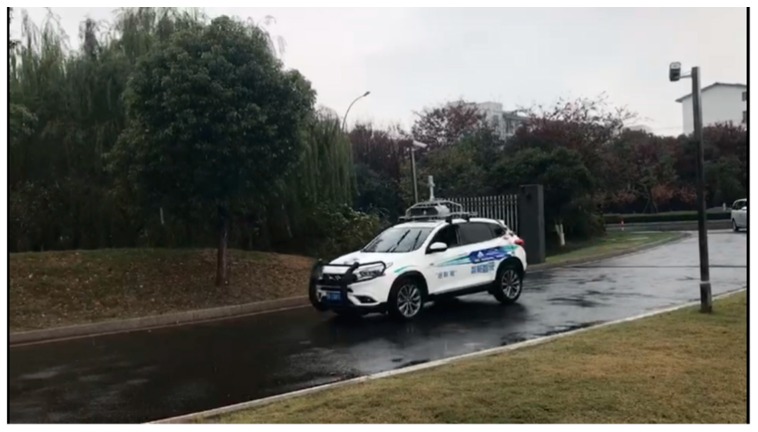
Platform of experiment: The “Tuyou” autonomous vehicle.

The proposed method was implemented in C and C++ as a multi-platform software, and was built and tested in the “Tuyou” autonomous vehicles shown in Figure 12. For the design and debugging stages of the project, we used an i7 core CPU 7567U @ 3.5 GHz with 8 GB RAM running Ubuntu 16.04. Sequences of road images were captured by a BFS-23S6C industrial camera, produced by the company of PointGrey, Richmond, BC, Canada, with a 12.5 mm focal length lens.

### 7.1. Performance of Our Platform

**Figure 13:**
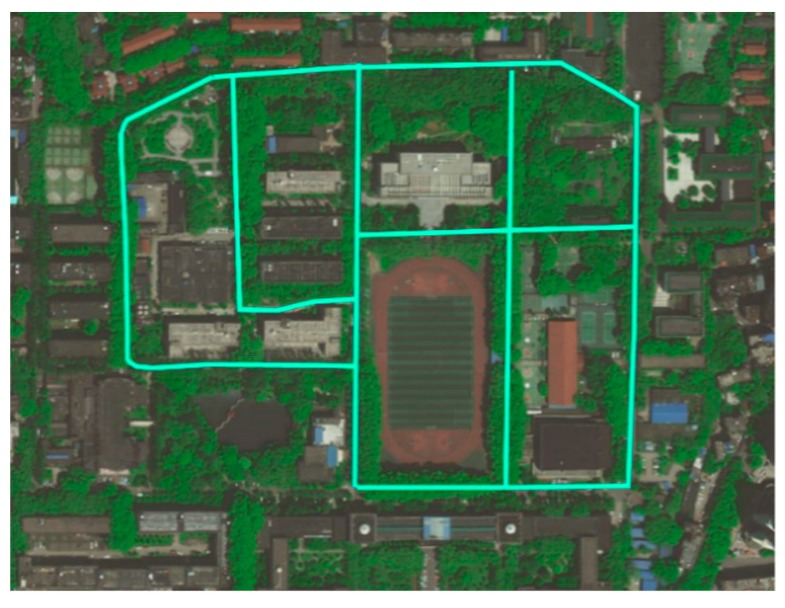
Experimental environment of our platform.

During the evaluation stage, it was necessary to test the algorithm on a real-time road environment using autonomous platforms, so we installed our program on an autonomous vehicle and combined it with a model predictive control (MPC) component to control the vehicle along the campus road in Wuhan University, Wuhan, China, as shown in Figure 13. The trajectory of motion planning was replaced with the middle line of both sides of the lane. The lane detection component in that system provided low-cost resolution for positioning the vehicle between both sides of the road. 

To ensure that the pixel width of the lane markings is clear and obvious, and the positions of the edge points could be located, the size of the input image was 1920 × 1200. As the width of the image was more than three times the proposed 640 × 480 and 320 × 240 resolutions, the linear relationship between the pixel width of the lane markings and their vertical coordinates in the image were robustly restricted. Additionally, some efficient methods were used in this paper in the feature extraction to speed up the algorithm. At first, the detection rows of the image were decided by projecting the rectangular grid on the road plane to the image plane which can reduce the size of input data. Then, the computation of the line segment voting process is related to the number of the edge points and insensitive to the size of the input image. As a result, although the input image was more than three times larger than with the traditional method, the elapsed time for our method was shorter and met the real-time requirements of the autonomous vehicle. 

**Figure 14:**
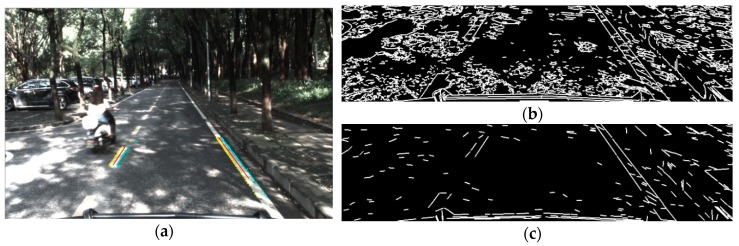
The size of the region of interest (ROI) was 1920 × 400 and elapse time of (**a**) the proposed method was 8 ms, (**b**) line segment detector (LSD) was 106 ms, and (**c**) fast line detector (FLD) was 36 ms.

As shown in Figure 14, the proposed method was compared to line segment detector (LSD) and fast line detector (FLD). The ROI was initialized in the first section. LSD and FLD algorithms were implemented with open source computer vision library (OpenCV), which ensured that the comparative experiment was not influenced by program language. The results of the experiment showed that the proposed method was nearly four times faster than the FLD algorithms. 

**Figure 15:**
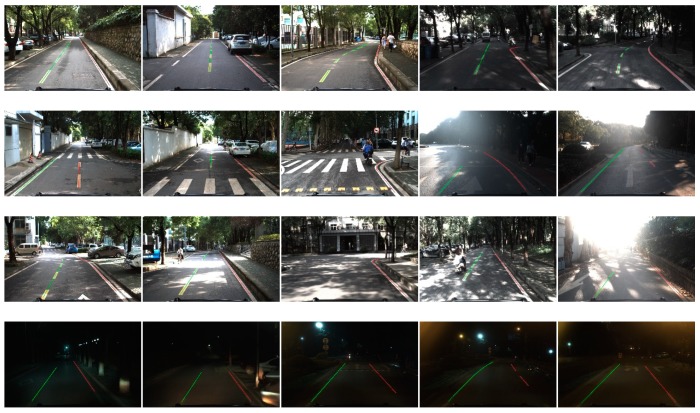
Some results of our method on a campus road, the left side of the current lane is colored with green and the right lane is red. The first row is in mild light conditions where the lane markings are constructed by straight line, polyline line or curb. The second row is in the intersections which contain zebra or arrow markings on the road. The third row was in bright light conditions which contain shadow, bright spot or backlighting. The last row consists of some night scenarios.

Table 1 shows the processing results of the algorithm in different road environments. The simple straight environment with mild light conditions requires the least time. Compared to the straight line scenario, the complex shape of the road will need extra computation to adjust width of the lane markings and fit the linear or nonlinear parts along the ego-lane, which lead to nearly 20%~30% increase of elapsed time. In addition, complex conditions like light spots, shadows, or other illumination could create noises, which were similar to lane markings, and would consume double the time of the simple straight environment. This is because these noise markings would add considerable computation for line segment detection and validation. Some results of our method are provided in Figure 15.

### 7.2. Performance on Public Database

**Figure 16:**
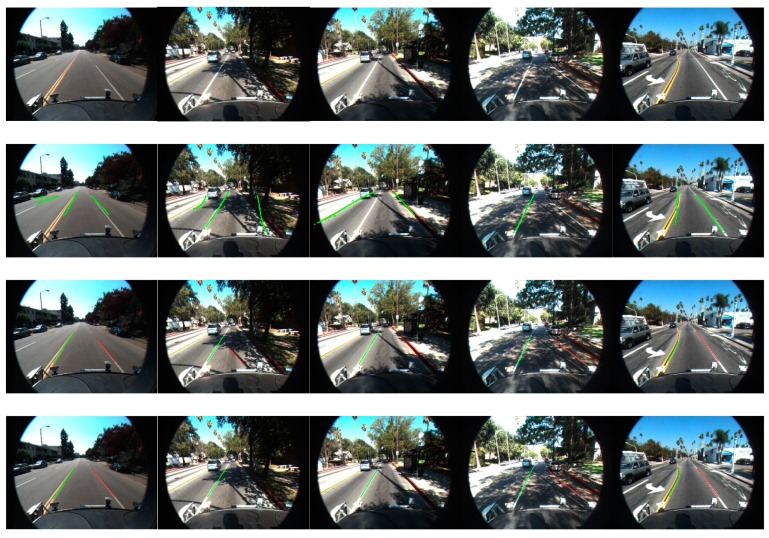
The first row shows the original images of the Caltech Database, the second row proposes the results of Aly’s method and the third row is the results of Niu’s method. The last row shows the results of our method.

In addition, the proposed method was tested on the public Caltech dataset. There are four image sequences (Cordova1, Cordova2, Washington1, and Washington2) in the Caltech dataset. However, the size of the images in the database are 640 × 480, which is three times smaller than our platform. The pixel width of lane markings in the far region was small, which led to the detection distance of that method being shorter than on our platform. Although adaptive problems are caused by the dataset, we also achieved good results because our method adopts a local adaptive algorithm. Table 2 shows the results of four scenarios in the Caltech dataset with a constraint on detection distance. According to the results of the experiment, the proposed method achieved better results except for in the Cordova2 scenario, because the lane markings in that image sequence contained several roads without continuous solid lines on both sides of the ego-lane. Figure 16 shows some results of the comparative experiments with Aly’s method [35], Niu’s method and our method on the dataset [40].

### 7.3. Limitations of Our Method

**Figure 17:**
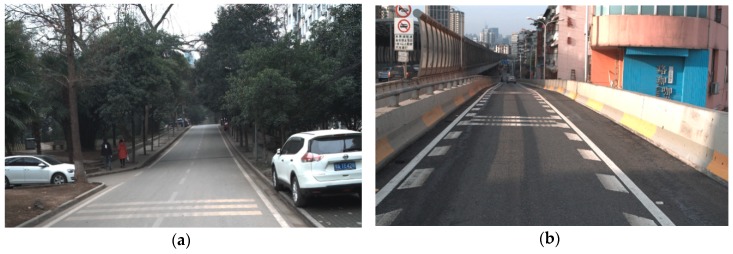
Limitation scenarios of our method with (**a**) upward and (**b**) downward slope.

We acknowledge that our proposed method has some limitations, the most serious of which is that the model selection method fails when the road scenario contains upward or downward slope, as shown in Figure 17. Although the lane markings in these pictures are obvious, and edge points can easily be extracted, the pitch angle between the camera and far area of the road has changed. The results of the position of the lane markings on the road plane will contain errors which can disturb the process of geometrical model selection and reduce the accuracy of tracking the model. The other limitation is that at least one side of the ego-lane should have continuous solid lines, which is widely used to extend the linear or nonlinear detection region and validate the line segments. However, as the number of road lanes is fewer than four in low-speed roads, a continuous solid line usually exists on one side of the ego-lane.

## 8. Conclusions

In this study, a robust lane detection method based on a monocular camera was proposed to handle complex conditions in a low-speed environment. The input image size for our method is high—up to 1920 × 1200—which is three times larger than other methods. By considering the error model of the vibration of the vehicle, our method provides the width of lane markings with high accuracy in the image plane. Compared to the traditional edge detection methods like, Sobel, Canny, Steer filter and symmetrical local threshold (SLT), the proposed method can extract the edge of the lane marking exactly and improve the robustness of the algorithm. Although the high-resolution images add the computation of the algorithm, the Bresenham line voting space (BLVS) proposed in this paper is insensitive to the increased pixels of the image and can provide a real-time line segment detection method. The piecewise fitting method combined with straight line, curve, and polyline ensures that the shapes of the road are adaptively described with high accuracy. Finally, two different Kalman filters were used to track key points in linear and nonlinear segments, which distinguishes the shapes of line segments and enhances the accuracy of the tracking of the geometrical model.

The proposed method is verified on our autonomous vehicle “Tuyou” and the public Caltech dataset, which contains complex road scenarios like intersections, bright spots and night. Experimental results show that the proposed method is robust and accurate. The computation of the proposed algorithm is different between the road scenarios, and even the most complex conditions also meet the real-time requirement.

## Figures and Tables

**Table 1:** Performance of our method in different scenarios.

Scenario	Number of Images	Elapsed Time (ms)	AR (%)	FN (%)
Straight line	5000	15.2	98.2	1.2
Curve	2500	20.6	95.6	2.6
Polyline	2000	18.8	97.5	1.8
Complex	4000	30.6	93.6	3.8

**Table 2:** Comparative experiment between Aly’s method [35] and our method.

Scenario	Number of Images	Aly’s Method [35]		Niu’s Method		Our Method	
		AR (%)	FP (%)	AR (%)	FP (%)	AR (%)	FN (%)
Cordova1	250	97.2	3.0	92.2	5.4	98.4	1.2
Cordova2	406	96.2	38.4	97.7	1.8	90.3	1.6
Washington1	337	96.7	4.7	96.9	2.5	97.2	2.8
Washington2	232	95.1	2.2	98.5	1.7	98.2	1.6
